# Adventitia Layer-Focused Microsurgical Flow Reconstruction for Long-Segment Tubular Stenosis of the Cervical Segment (C1) Internal Carotid Artery: Clinical Valuable Experience in 20 Cases

**DOI:** 10.3390/brainsci14030289

**Published:** 2024-03-19

**Authors:** Efecan Cekic, Mehmet Erkan Ustun

**Affiliations:** 1 Department of Neurosurgery, Polatli Duatepe State Hospital, Ankara 06010, Turkey; 2 Department of Neurosurgery, Private Clinic, Ankara 06010, Turkey; merkanustun@hotmail.com

**Keywords:** perivascular sympathectomy, cervical internal carotid artery, long-segment tubular stenosis, microsurgical intervention, tinnitus, migraine, transient ischemic attacks

## Abstract

To evaluate the efficacy of perivascular sympathectomy in managing adventitia layer-related long-segment tubular stenosis of cervical segment (C1) internal carotid arteries (ICAs) in a cohort where conventional medical and endovascular interventions were not viable options, we retrospectively analyzed 20 patients (8 males, 12 females, aged 41–63 years) who underwent perivascular sympathectomy for long-segment (>5 cm) tubular cervical ICA stenosis (non-atherosclerotic, non-intima related, and nondolichoarteriopathic) between 2017 and 2023. The procedure aimed to alleviate symptoms such as hemiparesis, pulsatile tinnitus, and migraines associated with transient ischemic attacks (TIAs). Preoperative and postoperative symptoms were assessed, and patient follow-up was conducted by MR angiography and perfusion studies. Postoperatively, 10 out of 11 migraine sufferers (90.9%) reported complete cessation of symptoms, while one patient (9.09%) experienced reduction in frequency and intensity. In cases of tinnitus, six out of nine patients (66.6%) reported complete resolution, two (22.2%) had reduced symptoms, and one (11.1%) saw no change. Regarding motor function, all 12 patients (100%) with initial hemiparesis (30–40% loss of motor function) showed complete recovery postoperatively. There was no TIA attack among the patients after the procedure in the mean two-year follow-up. Perivascular sympathectomy has shown promising results in alleviating symptoms and preventing recurrent cerebrovascular events in long-segment tubular stenosis of cervical ICAs.

## 1. Introduction

Managing cervical internal carotid artery (ICA) stenosis, a significant cause of cerebral ischemia, presents a complex clinical challenge [1]. A considerable portion of cerebral ischemic events are linked to abnormalities in the ICA, with a subset notably occurring within its cervical (extracranial) segment [2]. This condition’s prevalence, around 4% in the general population and increasing with age, calls for a multifaceted treatment [3]. While duplex sonography, computerized tomography (CT), and magnetic resonance (MR) angiography have emerged as the foremost diagnostic modalities, the treatment choice for cervical ICA stenoses varies, ranging from endovascular interventions and medical management to more invasive surgical options such as carotid endarterectomy [4].

In conventional treatments, carotid endarterectomy (CEA) and carotid artery stenting (CAS) have been pivotal in short-segment (<2–2.5 cm) pathologies of the ICA, which depend on atherosclerotic processes [5,6]. Randomized trials have established the efficacy of CEA in significantly reducing the 5-year risk of stroke in patients with symptomatic and asymptomatic stenoses [7].

However, some patients have long segments (>5 cm) of cervical ICA pathologies that are nonatherosclerotic and adventitia-related. These pathologies may be influenced by factors such as increased adventitia thickness and associated vascular remodeling, as indicated by research demonstrating the correlation between obesity, aging, and arterial structure changes. Increased adventitia thickness, which has been linked to heightened blood pressure and arterial stiffness, could contribute to such long-segment tubular stenoses by altering the mechanical properties and functional integrity of the artery, potentially impairing cerebral perfusion and enhancing susceptibility to vascular complication [8,9,10,11]. Standard interventions such as endovascular stenting may be less effective, prone to complications, or inappropriate. Patients usually present with symptoms such as hemiparesis, pulsatile tinnitus, migrainous attacks, and transient ischemic attacks (TIAs) [12,13] and find themselves at a therapeutic crossroads with limited options because of the long-segment tubular stenosis of the cervical ICA.

Our study elucidates a microsurgical approach tailored to twenty adventitia-related long-segment tubular cervical ICA stenosis (non-atherosclerotic, non-intima related, and nondolichoarteriopathic) cases. A subset of patients with tubular stenosis that did not responded to standard medical or feasible endovascular treatments were included. This precludes short-segment interventions and necessitates an innovative microsurgical approach. The senior author performed microsurgery as a perivascular sympathectomy for a long segment of tubular stenosis. This method aimed to enhance blood flow and alleviate ischemic symptoms, representing a crucial departure from standard treatment modalities. The surgical technique, avoiding the complexities of bypass-like procedures, is performed with greater applicability and lower morbidity risk, making it a preferable alternative in managing long-segment tubular stenosis. In contrast to procedures such as CEA and bypass surgeries, there is no requisite to halt blood flow during the operative intervention. We document each patient’s transition from preoperative discomfort to postoperative improvement, highlighting the potential of this microsurgical technique. Our findings affirm the feasibility and safety of this approach and demonstrate its effectiveness in enhancing patients’ quality of life.

## 2. Methods

In a retrospective study conducted between 2017 and 2023, we evaluated a cohort of 20 patients who underwent surgery for a long-segment tubular stenosis of the cervical ICA. These patients were selected due to drug resistance and were deemed unsuitable for endovascular treatment by interventionalists. All patients presented with symptoms associated with tubular stenosis, including hemiparesis, pulsatile tinnitus, migrainous attacks, and transient ischemic attacks (TIAs).

The inclusion criteria for this cohort were primarily based on a history of TIAs, with all patients having experienced at least one TIA episode. In this group, eleven patients had a history of migraine attacks and nine experienced tinnitus. Notably, the tinnitus was determined to be unrelated to otolaryngological conditions, and migraines were not associated with other neurological conditions. In our patient cohort, hemiparesis was specifically associated with long-segment tubular stenosis of the cervical ICA, a non-atherosclerotic and adventitia-related condition. To ensure a focused diagnosis, we conducted comprehensive brain and diffusion MRI scans on all patients to rule out other common causes of hemiparesis, such as tumors and vascular lesions. Additionally, clinical evaluations did not reveal signs that would suggest radiculopathy indicative of cervical disk pathology or findings consistent with thoracic outlet syndrome, further reinforcing the association between hemiparesis and the observed stenosis. This rigorous diagnostic approach allowed us to exclude other potential etiologies and affirm the link between hemiparesis and structural arterial changes in our study population.

**Figure 1:**
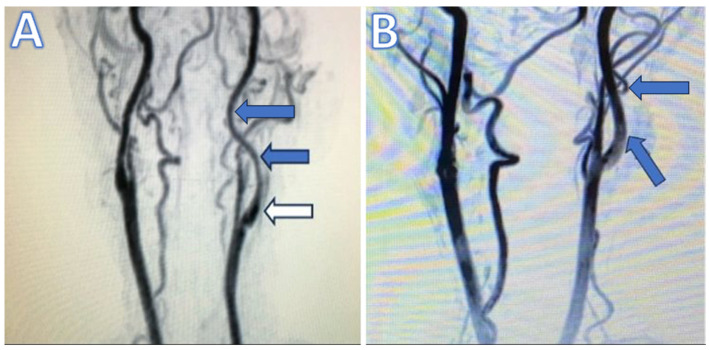
(**A**) Preoperative magnetic resonance (MR) angiography illustrating long-segment tubular stenosis. Blue arrows point to the tubular stenotic sections, while white arrow indicates the unaffected carotid bulb. (**B**) Postoperative MR angiography in the first month shows the dilatation of the internal carotid artery (blue arrows) compared to the preoperative MR angiography.

Patients were primarily assessed using MR and/or CT angiography. Our selection process and criteria were particularly underscored by the presence of a first segment, which is the cervical segment (according to Ziyal [14] and Bouthillier [15]) of ICA long-segment tubular stenosis observable on cervical MRI angiography, which starts from the aorta, strictly within the realm of tubular stenosis that is not eligible for endovascular treatment, thus aligning with our targeted focus and diagnostic precision, as shown in Figure 1. This segment is referred to as ‘C1’ following the classifications by Ziyal et al. [14] and Bouthillier et al. [15], which is not to be confused with the ‘C1’ segment in the Fischer classification, where ‘C1’ represents the intracranial segment from the ICA’s termination to the origin of the posterior communicating artery. Hence, the Fischer classification is not considered in our study of the cervical ICA segment (C1) [16].

We meticulously measured the lengths of the cervical ICA tubular stenoses. Detailed analyses of preoperative and postoperative carotid artery thicknesses allowed us to calculate the degree of stenosis using MR and/or CT angiography.

These imaging modalities were instrumental in identifying the extent and nature of the stenosis and in ruling out those with atherosclerotic or dolichoarteriopathic lesions. Regarding immunological tests, most of the patients had a panel of tests, including but not limited to ANA, ESR, CRP, and specific markers when vasculitis or other inflammatory diseases were suspected, to exclude patients with inflammatory conditions. Besides that, every patient in this study has a perfusion CT or MR image for the possibility of hypoperfusion areas. Patients were followed up postoperatively at intervals of 1, 6, 12, and 24 months, with an average follow-up period of one and half years, for comprehensive assessment and monitoring of clinical outcomes.

Patients with anatomical variations indicative of dolichoarteriopathies were excluded. Additionally, we omitted individuals with prior carotid interventions, acute dissections, inflammatory conditions like vasculitis, intima-related pathologies such as atherosclerosis, post-radiation arterial changes, and recent cerebrovascular events to maintain a focused evaluation of tubular stenosis exclusive of these confounding factors. In our study, C2–C5 segments were not included since we did not encounter any instances of tubular stenosis in the petrous or subsequent segments of the ICA.

## 3. Surgical Technique

All surgical procedures were performed primarily by the senior author (M.E.U). Informed consent was obtained from all patients, and the ethics committee approved this study, documented under the approval number 2/12, dated 30 March 2022.

**Figure 2:**
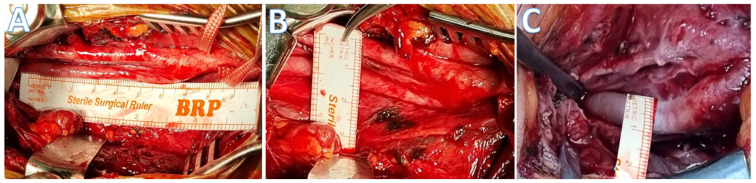
(**A**,**B**) The perioperative micrographic representation delineates extensive, long-segment tubular stenosis of the cervical segment of the internal carotid artery. (**C**) The perioperative post-interventional microscopic imagery reveals vascular dilation after perivascular sympathectomy, indicating a successful therapeutic response to the procedure.

Our surgical intervention aimed to alleviate the hemodynamic compromise associated with long-segment tubular stenosis of the internal carotid artery, as depicted in Figure 2. Access to the carotid vasculature was facilitated through a strategically placed curvilinear incision extending from the angle of the mandible to the cervical region, delineating the carotid bifurcation. The dissection was meticulously advanced to isolate the common carotid artery and further delineate both the external and internal carotid arteries. Notably, the external carotid artery presented with a caliber equal to or exceeding that of the internal carotid artery, an observation crucial for preoperative planning. The pathological segment, characterized by stenosis, was exposed over its entire length, necessitating extensive dissection to enable comprehensive treatment. The surgical strategy included the administration of papaverine to effectuate vasodilation, followed by a precise sympathectomy along the afflicted segment. This approach is called perivascular sympathectomy [17]. Upon exposure of the internal carotid artery and during the perivascular sympathectomy, a thickened fibrotic adventitial layer was observed. Perioperatively, before the intervention, the arterial pulsation was almost invisible; however, following the intervention, we observed a significant resurgence in pulsation and dilation of the artery, indicative of the re-established blood flow and procedural success in different instances. In this study, all patients exhibited a notable characteristic: the carotid bulb segment was consistently intact and free from stenotic changes. The stenoses observed in each case were localized distal to the carotid bulb, as depicted in Figure 2.

## 4. Results

This retrospective study scrutinized the clinical efficacy of perivascular sympathectomy in 20 patients suffering from long-segment tubular stenosis of the cervical ICA. The primary aim was to address the symptomatology and subsequent quality-of-life improvements post-surgery.

The lengths of the cervical ICA tubular stenosis treated varied from 5.2 to 9.9 cm, averaging around 7.3 cm across the cohort. The mean carotid thickness prior to surgical intervention was measured between 2.5 and 4.3 mm, with an average of 3.6 mm. Postoperatively, the carotid thickness showed a significant increase, ranging between 6.2 and 9.1 mm, reaching an average of 7.4 mm. This represents a substantial expansion of the carotid lumen following the surgical procedure. These measurements were crucial for assessing the severity of the condition and the effectiveness of the microsurgery applied.

Upon postoperative evaluation, migraine cessation was reported in 90.9% (10/11) of the patients, with the remaining 9.1% (1/11) experiencing a substantial reduction in both the frequency and intensity of the episodes. Tinnitus, initially present in 45% (9/20) of the cohort, showed complete resolution in 66.6% (6/9) of the cases. The remaining patients reported varied degrees of symptomatic relief, with 22.2% (2/9) noting a decrease in symptoms and one patient observing no change.

Motor function recovery was remarkable, with a 100% (12/12) recuperation rate in patients with hemiparesis of 30–40% strength loss. It is notable, however, that one individual developed apraxia, notwithstanding the recovery of motor capabilities.

Critically, no TIA episodes were reported in the entire patient group during the mean follow-up period of two years post-procedure. This indicates a significant postoperative decline in the recurrence of TIAs, suggesting the surgical intervention’s effectiveness in mitigating the risk of further cerebrovascular events.

These results affirm the therapeutic potential of perivascular sympathectomy for patients with cervical ICA stenosis, providing substantial symptom relief and enhancing postoperative cerebrovascular health, as shown in Table 1. The findings advocate for its consideration as a viable treatment option, especially in cases where traditional medical and endovascular interventions are deemed unsuitable.

## 5. Discussion

Tinnitus, often a symptom associated with otological conditions, can sometimes manifest vascular abnormalities [18]. In our study, the resolution of tinnitus in most patients post-microsurgery suggests a vascular origin, possibly due to altered hemodynamics within the carotid system. This observation is aligned with previous reports, which have indicated that ICA pathology (especially the petrous segment, because the closest nest to the inner auditory bones can manifest as pulsatile tinnitus) is further substantiated by the improvement following vascular interventions [19]. The existing academic discourse acknowledges instances of tinnitus arising from stenoses in the supraclinoid segment of the internal carotid artery, albeit less frequently reported [20]. In the literature, tinnitus associated with ICA stenosis is often attributed to turbulent flow across a localized narrowing, which generates a perceptible sound [21]. One study investigates hemodynamic variations in pathologically kinked ICA (PK ICA) in patients with fibromuscular dysplasia (FMD) and hypertensive heart disease. Utilizing Doppler ultrasound, it was found that hemodynamic parameters in PK ICA due to FMD showed a decrease in blood flow velocity when patients were standing, suggesting more pronounced hemodynamic impairment in hypertensive heart disease. S-shaped kinks significantly increased blood flow turbulence, indicating that the position of ICA kinking is critical for detecting changes in blood flow dynamics [22]. Another study identifies tortuosities and coiling of the ICA as factors associated with pulsatile tinnitus. This condition is believed to be caused by altered hemodynamics and turbulent blood flow within the artery. Treatment focuses on correctly identifying these vascular anomalies via computed tomography (CT) angiography, essential for effective management and differentiation from other vascular pathologies [23].

However, in our patient cohort, the pathophysiological underpinnings deviate from this commonly accepted mechanism due to the unique presentation of long-segment tubular stenosis. Our findings suggest that the tinnitus in these cases may stem from a “jet flow” phenomenon induced by the transition of blood flow from a broad to a narrowed, elongated segment. Moreover, the proximity of this tubular stenosis to the petrous portion of the ICA adjacent to the bony structures of the ear likely exacerbates the auditory symptoms. Despite originating in the first segment of the ICA, this tubular stenosis extends sufficiently to impact areas closer to the acoustic apparatus, thereby producing tinnitus. This hypothesis contributes to the existing discourse on vascular tinnitus. It underscores the need for a tailored approach in assessing and managing patients presenting with this symptom in the context of carotid artery disease.

We observe a parallel in the migraine episodes experienced by patients with ICA stenosis, which we posit to stem from a flow diversion or steal phenomenon towards the external carotid artery (ECA). This is akin to findings by Herial et al. and Kimura et al., who describe headaches and facial pain resulting from altered hemodynamics due to significant ICA stenosis [24,25]. Our clinical data suggest that similar to the flow-diversion headaches detailed by Herial et al., the migrainous attacks in our patients may be attributed to excessive blood diversion to the ECA due to tubular stenosis in the ICA. These observations underscore the potential of vascular abnormalities in contributing to migraine pathophysiology and highlight the need for further research into the mechanisms linking ICA stenosis with neurovascular headaches. In our cohort, we noted that patients with tubular stenosis experienced considerable benefits from the surgical approach in terms of migraine attack frequency and intensity. This suggests the importance of surgical intervention in cases of tubular stenosis and underscores the necessity of vascular assessment in migraine patients, given the potential for underlying vascular pathologies within the carotid system.

In our cohort of 20 patients, all had experienced TIAs before surgical intervention—8 patients reported a single episode, while 12 reported multiple events. This clinical presentation is consistent with the narrative review by Pini et al., which underlines the urgency of addressing symptomatic ICA stenosis to prevent recurrent ischemic events [26]. Following the insights presented by Siddiqui et al., the link between ICA stenosis and TIAs is well established, with ICA revascularization procedures like stenting serving as critical interventions for stroke prevention [27]. Another study assessed the efficacy of surgical revascularization for symptomatic kinking of the ICA. The procedure, involving ICA transaction and end-to-side anastomosis, was applied to 25 patients with a history of TIA or stroke, resulting in no early postoperative strokes or deaths and a very low incidence of complications. Long-term follow-up showed favorable outcomes, with minimal recurrent stenosis or stroke cases, highlighting the procedure’s safety and effectiveness [28]. Unlike other studies, we addressed adventitia-related long-segment tubular ICA stenosis that was not amenable to endovascular treatment or was resistant to medical treatment. Employing perivascular sympathectomy, the complete cessation or substantial reduction in TIA episodes post-surgery indicates the effectiveness of this treatment modality.

**Figure 3:**
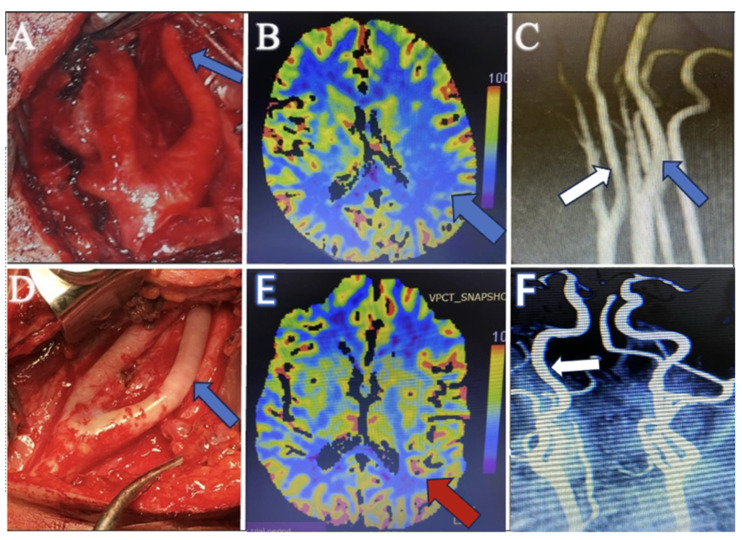
(**A**) Perioperative surgical microscope view before intervention demonstrates the long-segment tubular stenosis of the internal carotid artery (blue arrow). (**B**) Preoperative perfusion CT image shows the hypoperfusion area in the paracentral lobule (blue arrow), which explains the patient’s TIA attacks and hemiparesis. (**C**) The preoperative MR angiography image shows the internal carotid artery (white arrow) having a similar diameter to the external carotid artery. However, it appears narrower than the internal carotid artery on the opposite side (blue arrow). (**D**) Perioperative surgical microscope view after perivascular sympathectomy, which shows the dilatation of the internal carotid artery (blue arrow). (**E**) Postoperative perfusion CT images demonstrate the perfusion restoration (red arrow), which is compatible with postoperative improvement. (**F**) Postoperative MR angiography shows the dilatation of the internal carotid artery (white arrow).

Hemiparesis, observed in conjunction with other conditions, improved significantly in all patients postoperatively. This reinforces the importance of adequate cerebral perfusion and the role of ICA tubular stenosis in cerebral ischemic events. While recognized, the link between stenosis and motor deficits has been underscored by our findings, which demonstrate marked postoperative neurological recovery. In our study, patients with hemiparesis associated with migraines and TIAs underwent perfusion images. In eight of the ten affected patients, areas of hypoperfusion that could contribute to these symptoms were identified despite the lack of findings in standard MRI scans. This suggests that perfusion imaging can reveal cerebral blood flow deficits not apparent in conventional MR images pre- and postoperatively, as depicted in Figure 3.

During the surgical exploration for managing carotid artery stenosis, an abnormal observation was made concerning the relative calibers of the carotid vessels. When evaluating MR angiographies, it is essential to compare the ICA with the ECA on the same side and the ICA on the opposing side to ensure that long-segment tubular stenosis is not missed. In cases where MR angiography reveals similar calibers between the ICA and ECA, surgical findings demonstrate a notably narrower ICA than the ECA, and a detailed assessment, including clinical presentation and additional MR modalities, such as perfusion studies, becomes crucial. This comprehensive approach is necessary to establish a diagnosis of tubular stenosis, especially when imaging findings may not fully correlate with intraoperative observations, as depicted in Figure 3. Therefore, heightened vigilance is imperative to ensure that such pathologies are noticed.

In our patient cohort, the notable absence of ICA tortuosity contrasts with its known association with connective tissue diseases, as reported by Welby et al., who observed a significant prevalence in conditions such as Marfan and Loeys–Dietz syndromes [29]. Our findings of tubular stenosis suggest pathology originating from the adventitia layer of the vessel, potentially linked to conditions like genetic disorders outlined in the literature. This insight may guide future evaluations of vascular anomalies, especially when classical signs of connective tissue disease are absent.

Our study addresses lesions exceeding 5 cm. The adventitia layer is the principal site of pathology, influenced by aberrant sympathetic activity. There was no intima-related pathology. This evidence supports our rationale for selecting open surgery to resolve vasospasm and restore function directly. This strategy may offer a definitive solution for long-segment tubular ICA stenosis. The microsurgical method seeks to circumvent this by instantly relieving vasospasms, thus contributing to a functional resolution not typically achievable through endovascular treatment alone. This approach reduces the increased risk of stent migration, thrombosis, and occlusion seen in the conjoined stent technique, which may be appropriate for long-segment radiation-induced ICA stenosis [30,31]. Complications are lower with single-stent placements than with multiple-stent applications and are even less common in CEA [32,33,34].

Despite being a minimally invasive procedure compared to CEA and microsurgery, CAS can also present significant complications. Most complications arise during the procedure, while others may develop within the first seven days postoperatively. These can include stroke and TIAs, bradycardia, persistent hypotension, and hyperperfusion syndrome. In addition, certain anatomical features can pose a high risk in CAS procedures. These include the type of aortic arch (type 2, type 3, and bovine-type arch), being over the age of 80, recent symptomatic patients, clopidogrel allergy, resistance to aspirin or clopidogrel, severely tortuous ICA arteries, proximal ICA pathologies, concentric calcification, hemorrhagic plaques, intraluminal thrombus, preocclusive stenosis, and peripheral severe arterial disease that impedes access to the carotid arteries, all of which constitute a high-risk group [35].

A vital issue encountered during follow-up after the CAS procedure is stent restenosis. The literature reports higher restenosis rates in covered stents compared to bare-metal stents. Neointimal hyperplasia is the cause of stent restenosis, which develops due to physical irritation between the vessel and the stent, endothelial dysfunction, and chronic inflammation [36].

The high incidence of stent restenosis in covered stent applications is attributed to the covered stent’s prevention of endothelialization and contractions at the stent’s distal and proximal ends, which are other disadvantages. In another study by Schillinger et al., comparing covered and bare-metal stent therapies, the trial had to be terminated due to a 38% restenosis rate occurring within the first six months after covered stent implantation. This study also emphasizes that despite the high rate of restenosis with covered stent usage, it is highlighted that there is a reduction in the risk of microemboli [37].

Our minimally invasive surgical technique can avoid many adverse complications and undesirable outcomes. Additionally, while short-segment carotid narrowing can be treated with a single stent, long-segment narrowing (>5 cm) may require long stents with lower radial force or multiple stent deployments. Increased stent length can reduce the stent’s radial force and adherence, leading to complications such as migration, incomplete expansion, and embolic events. It has been shown that applying multiple overlapping stents can create unwanted complications such as microemboli and stent migration [38].

Additionally, there are two main types of stents: self-expandable and balloon-expandable. Balloon angioplasty is required to enlarge the carotid lumen in both stent applications. In our patient group, since the pathology is not intravascular but an increase in vasospasm activity on the outside layers of the vessel, the intervention performed with angioplasty may lead to an increase in sympathetic activity and a further narrowing of the vessel lumen. Our patient cohort experienced low temporary morbidity, such as swallowing difficulties and hoarseness.

Our study provides preliminary evidence of the efficacy of perivascular sympathectomy in treating long-segment cervical ICA stenosis. However, there are limitations to consider. The sample size of 20 patients, while sufficient for an initial investigation, is relatively small, and more extensive multicenter studies are needed to validate our findings. Additionally, the retrospective nature of this study may introduce selection bias, and prospective studies could offer more robust data.

Future research should also establish standardized protocols for the surgical technique and compare the long-term outcomes of perivascular sympathectomy with traditional treatment modalities. Incorporating cutting-edge imaging modalities, applying intraoperative machine learning algorithms powered by artificial intelligence, and synthesizing functional neurological evaluations before and after surgical interventions are poised to revolutionize our understanding and optimize surgical processes and patient prognoses [39]. Ultimately, these future directions could lead to more personalized treatment strategies for patients with complex vascular pathologies.

## 6. Conclusions

Perivascular sympathectomy to treat long-segment tubular cervical ICA stenosis demonstrated significant improvement in patients for whom conventional medical and endovascular treatments were not an option. This approach has proven to be a viable and effective alternative, emphasizing the importance of individualized treatment strategies in complex vascular conditions. It underscores the critical role of a comprehensive diagnostic evaluation, incorporating MR angiography and perfusion studies, especially when clinical symptoms of cerebrovascular insufficiency are present. This study underscores the necessity for a tailored surgical approach and advocates for more extensive prospective studies to confirm these valuable experiences of preliminary findings.

## Figures and Tables

**Table 1:** Comparative analysis of preoperative and postoperative symptoms in patients who underwent microsurgery for long-segment cervical internal carotid artery stenosis.

Patient Group	Complete Resolution	Partial Improvement	No Change
Migraine (*n* = 11)	10 (90.9%)	1 (9.1%)	0 (0%)
Tinnitus (*n* = 9)	6 (66.6%)	2 (22.2%)	1 (11.1%)
Hemiparesis (*n* = 12)	12 (100%)	0 (0%)	0 (0%)
TIA attacks (*n* = 20)	20 (100%)	0 (0%)	0 (0%)

## Data Availability

The data supporting the findings of this study, including all images and datasets, are available from the corresponding author, Efecan Cekic, upon reasonable request. No additional external data repositories were used, and the data are not publicly archived due to the nature of this study. All relevant data are contained within the article.
